# Supplementary material for: Leukaemia exposure alters the transcriptional profile and function of BCR::ABL1 negative macrophages in the bone marrow niche
Source: Nat Commun. 2024 Feb 5;15:1090. doi: 10.1038/s41467-024-45471-0 (PMC10844594; doi:10.1038/s41467-024-45471-0)

Leukaemia Exposure Alters the Transcriptional Profile and Function of BCR::ABL1 Negative Macrophages in the Bone Marrow Niche

Amy Dawson[1*], Martha M. Zarou[1*], Bodhayan Prasad[1], Joana Bittencourt-Silvestre[2], Désirée Zerbst[1], Ekaterini Himonas[1], Ya-Ching Hsieh[3], Isabel van Loon[1], Giovanny Rodriguez Blanco[3], Angela Ianniciello[1], Zsombor Kerekes[1] Vaidehi Krishnan[4], Puneet Agarwal[5], Hassan Almasoudi[1,6], Laura McCluskey[2], Lisa E. M. Hopcroft[2], Mary T. Scott[1], Pablo Baquero[1,7], Karen Dunn[2], David Vetrie[1], Mhairi Copland[2], Ravi Bhatia[5], Seth B. Coffelt[1,3], Ong Sin Tiong[4] , Helen Wheadon[2], Sara Zanivan[1,3], ∞Kristina Kirschner[1,3], ∞G. Vignir Helgason[1]

[1]Wolfson Wohl Cancer Research Centre, School of Cancer Sciences, University of Glasgow, Garscube Estate, Switchback Road, Bearsden, G61 1QH, UK. [2]Paul O'Gorman Leukaemia Research Centre, School of Cancer Sciences, University of Glasgow, Gartnavel General Hospital, 21 Shelley Rd, Glasgow G12 0ZD, UK. [3]Cancer Research UK Scotland Institute, Garscube Estate, Switchback Road, Bearsden, Glasgow, G61 1BD, UK. [4]Cancer & Stem Cell Biology Signature Research Programme, Duke-NUS Medical School, Singapore. [5]Division of Hematology and Oncology, Department of Medicine, University of Alabama at Birmingham, Birmingham, Alabama, USA. [6]Department of Clinical Laboratory Sciences, College of Applied Medical Sciences, Najran University, Najran 61441, Kingdom Saudi Arabia. [7]Universidad de Alcalá, Facultad de Medicina y Ciencias de la Salud, Dpto. de Biología de Sistemas, Unidad de Bioquímica y Biología Molecular, E-28805 Madrid, Spain. *These authors contributed equally to this work.

Corresponding authors. Kristina.Kirschner@glasgow.ac.uk Vignir.Helgason@Glasgow.ac.uk

Supplementary Information File containing:

Supplementary Figures 1-15 (and Figure Legends)

Source Data (in separate files)

Supplementary Tables 1-4

Full Western Blot membranes (uncropped and unprocessed scans of the western blots shown in the supplementary figures and Source Data File)

Supplementary Fig. 1

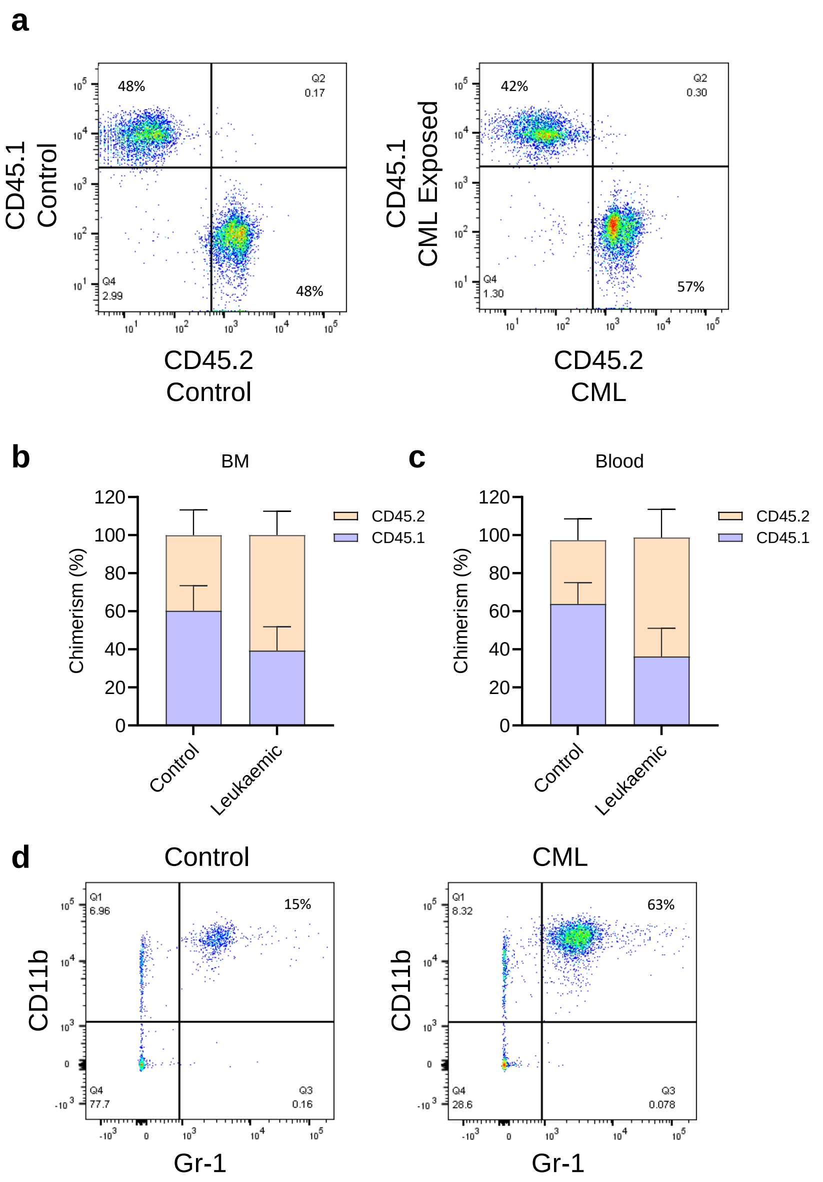

Supplementary Figure 1: Analysis of bone marrow (BM) and blood of chimeric mice after BCR-ABL induction. **a:** Representative flow cytometry plot of chimerism in BM. **b-c:** Chimerism in BM (b) and blood (c) at experimental endpoint. **d:** Representative flow cytometry plots of myeloproliferation in BM.

Supplementary Fig. 2

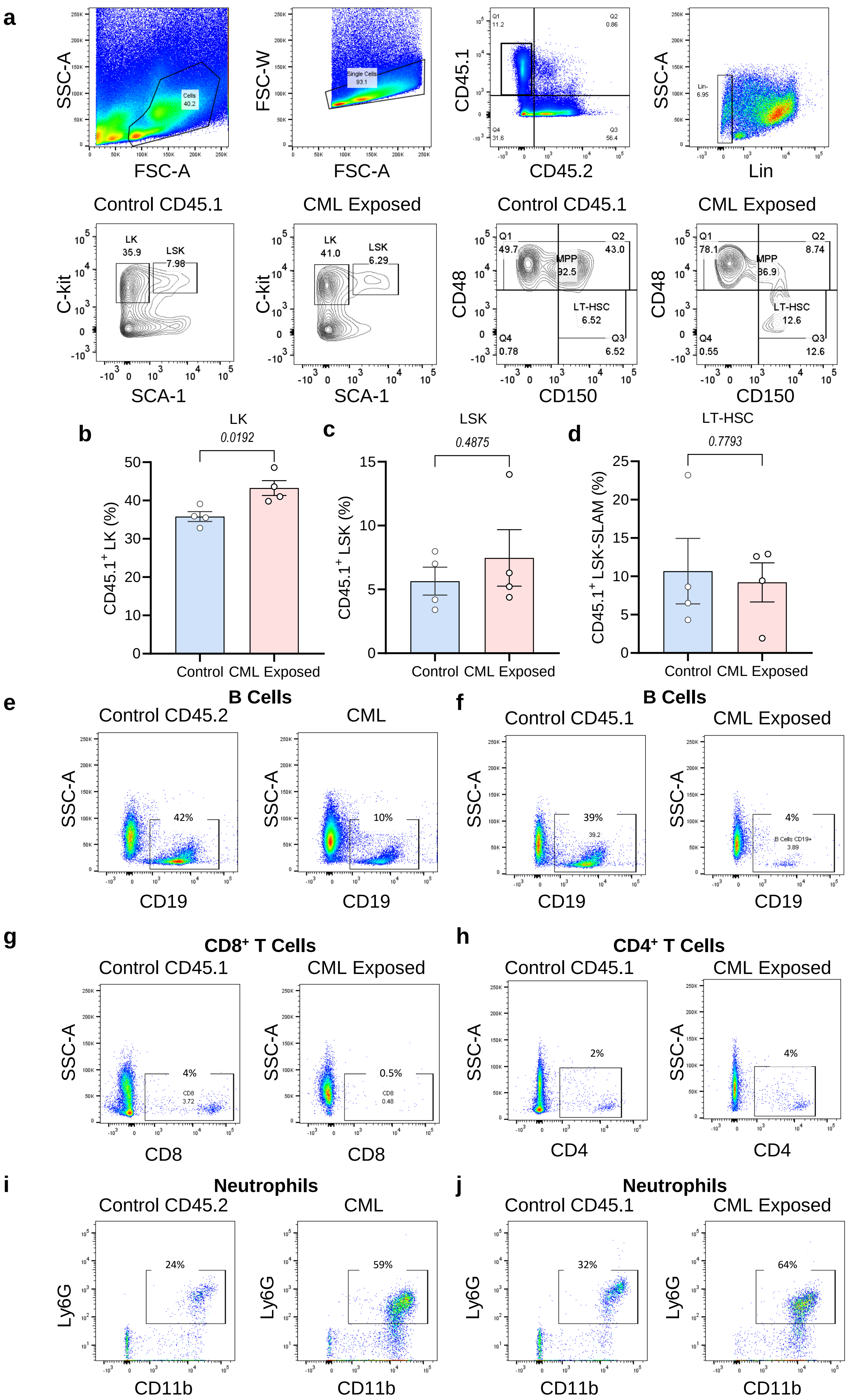

Supplementary Fig. 2 cont.

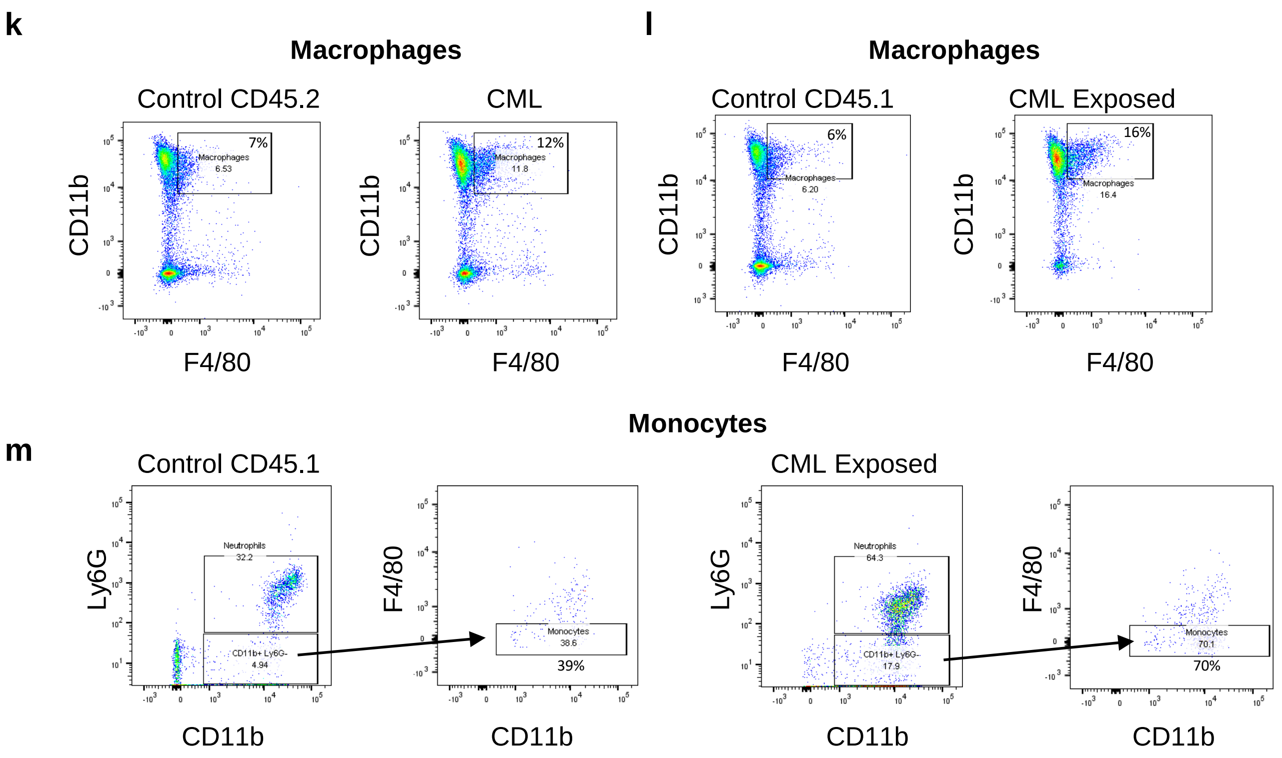

Supplementary Figure 2: Gating strategy and flow cytometry analysis of BM located myeloid cells and lymphocytes. a: Representative gating strategy for isolation of Lin$^-$ c-Kit$^+$ (LK) and Lin$^-$ c-Kit$^+$ Sca-1$^+$ and Lin-c-Kit$^+$ Sca-1$^+$ CD150$^+$ CD48$^-$ (LT-HSC) cells in CD45.1 BM. **b-d**: Quantification of LK (**b**), LSK (**c**) and LT-HSC (**d**) populations (%) of parent population (n=4 mice per experimental arm). **e-m**: Representative flow cytometry plots of CD19$^+$ B cells (**e-f**), CD8$^+$ T cells (**g**), CD4$^+$ T cells (**h**), CD11b$^+$Ly6G$^+$ cells (**i-j**), CD11b$^+$F4/80$^+$ cells (**k-l**), and CD11b$^+$Ly6G$^-$F4/80$^-$ cells (**m**). Data are shown as the mean ± s.e.m. P-values were calculated using unpaired two-tailed t-test (**b-d**).

Supplementary Fig. 3

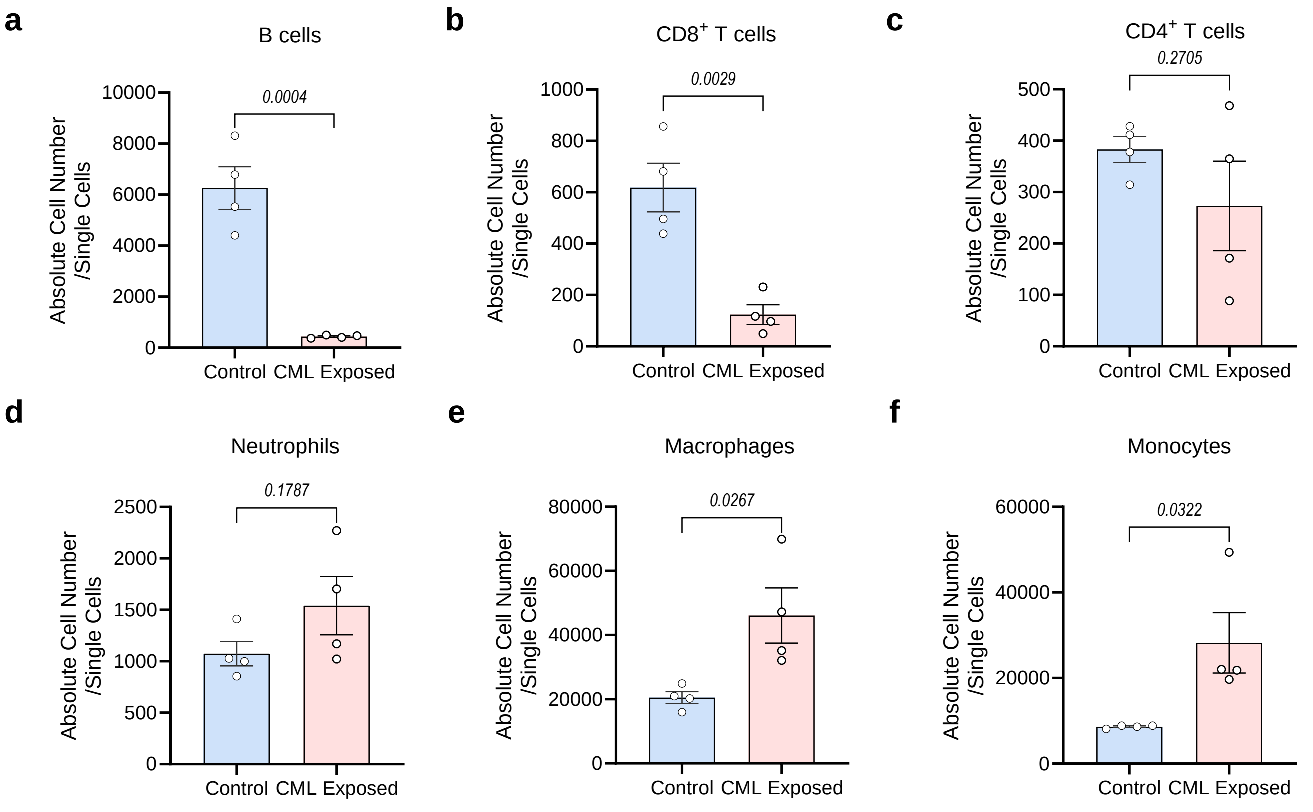

Supplementary Figure 3: Absolute cell numbers of control and CML exposed BM cell populations. Absolute cell numbers in BM in CD45.1 fraction of chimeric control or CML mice (n=4 mice per experimental arm). Data are shown as the mean ± s.e.m. P-values were calculated using unpaired two-tailed t-test (**a-f**).

Supplementary Fig. 4

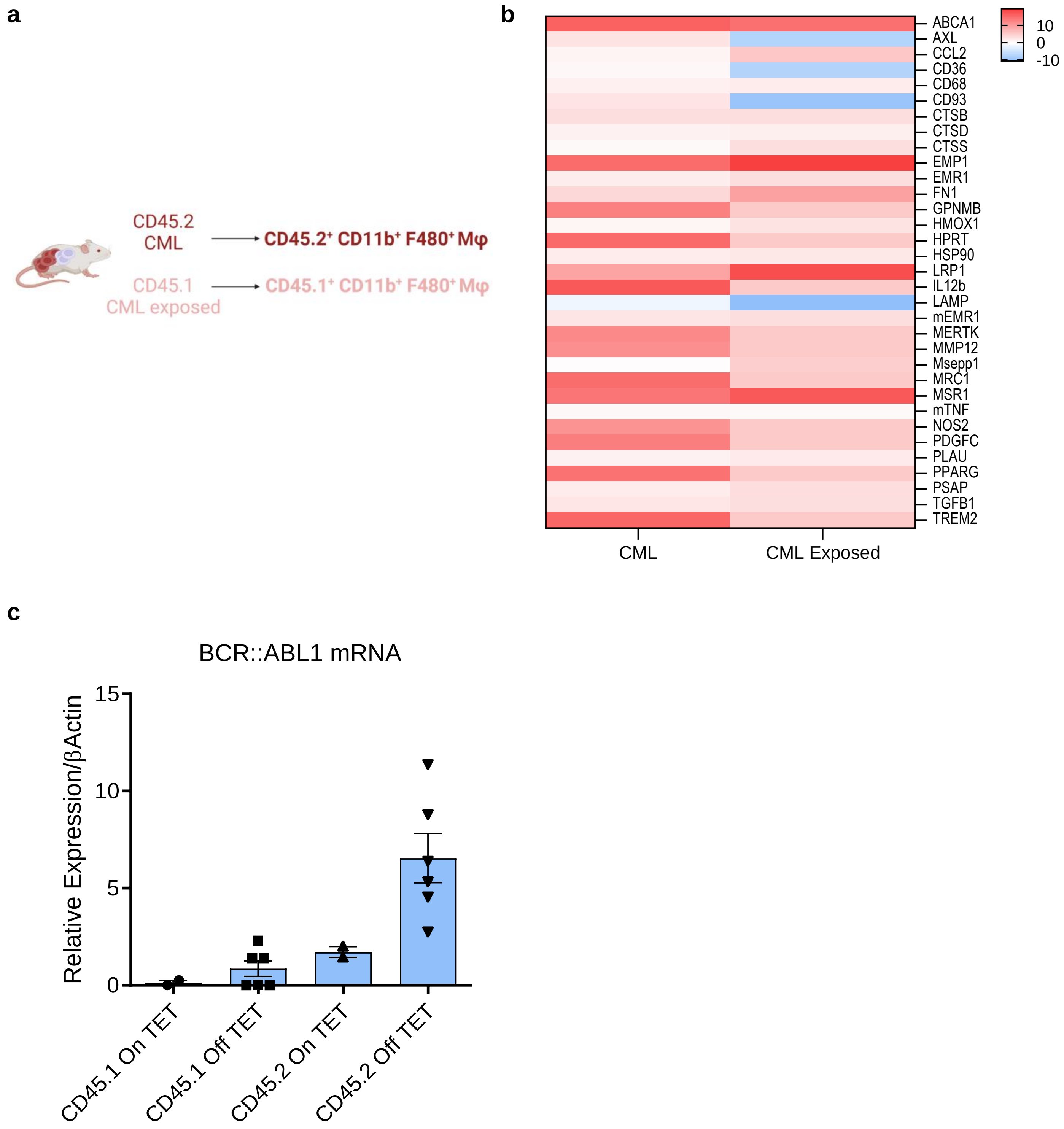

Supplementary Figure 4: CML exposed and CML macrophages display unique gene expression profiles. a-b: Schematic outline of experimental design (**a**) and heatmap (**b**) of Fluidigm gene expression analysis of CD11b$^+$F4/80$^+$ macrophages sorted from CD45.2 (CML) or CD45.1 (CML exposed) BM (n = 4 mice). Schematic created with Biorender.com (Agreement number FR268HVD0N). **c**: RT-qPCR mRNA expression of BCR-ABL p210 in CD11b$^+$ F4/80$^+$ cells isolated from CD45.1 and CD45.2 BM fractions of mice maintained on or off tetracycline (TET) for 15 days (n=2 mice for CD45.1 on TET and CD45.2 on TET, n=6 mice for CD45.1 off TET and CD45.2 off TET).

Supplementary Figure 5

a

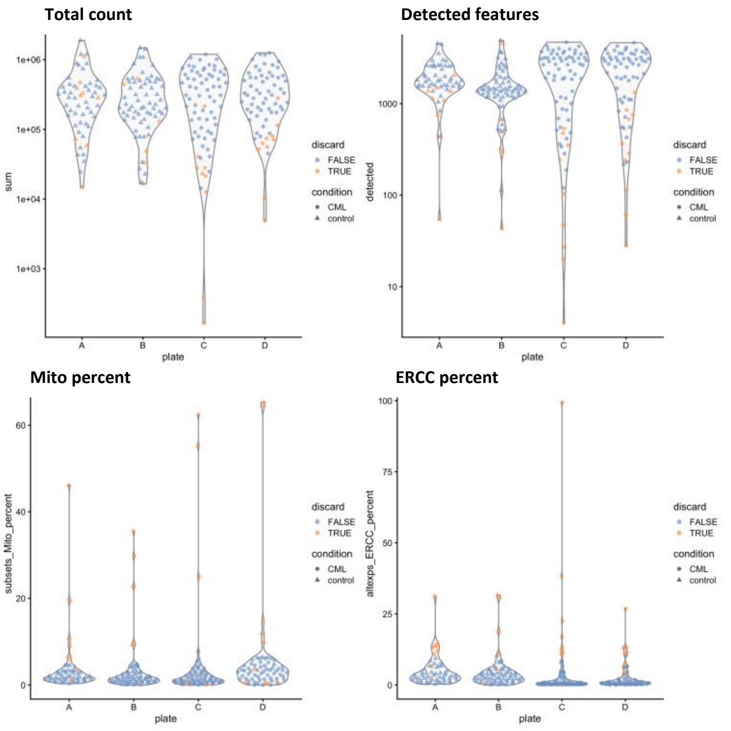

b

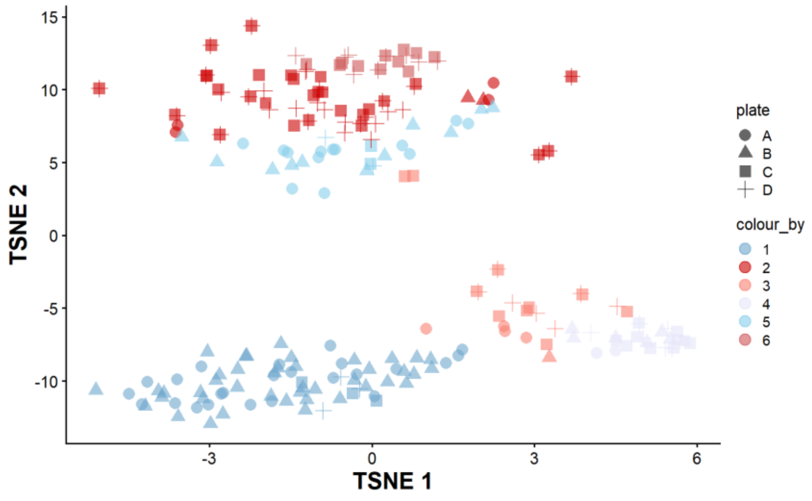

Supplementary Figure 5: sc-RNAseq QC plots. a: Quality control analysis of scRNAseq data showing total counts, features, mitochondrial and ERCC content. Orange denotes cells excluded from further analysis for not passing the QC threshold. ERCC threshold of 10.539%, mitochondrial content threshold of 8.433%, sum of counts threshold of 5137.281 and feature number threshold of 96.785. **(b)** t-SNE plot showing cell clustering according to plates processed.

Supplementary Fig. 6

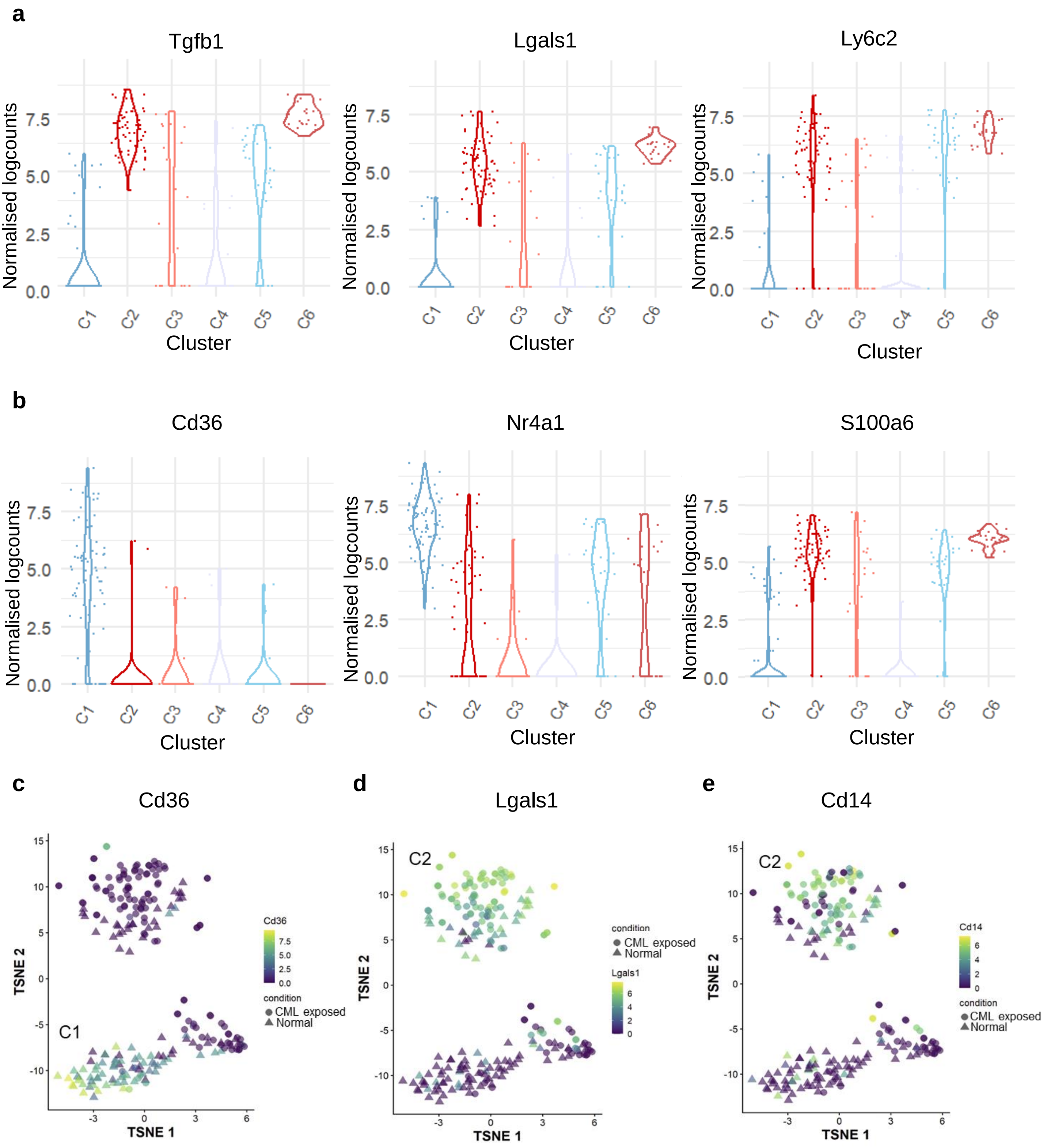

Supplementary Figure 6: CML Exposed Macrophages Form Unique Subpopulations with distinct transcriptional profiles. a-b: Violin plots of normalised log counts of *Tgfbi*, *Lgals1*, *Ly6c2* (**a**), *Cd36*, *Nr4a1* and *S100a6* (**b**). **c-e**: Marker panel identification of single cell CD45.1^{+} CD11b^{+} F4/80^{+} RNA sequencing utilising COMET analysis. **c:** Cluster 1 *Cd36* enrichment. **d:** Cluster 2 *Lgals1* enrichment. **e:** Cluster 2 *Cd14* enrichment.

Supplementary Fig. 7:

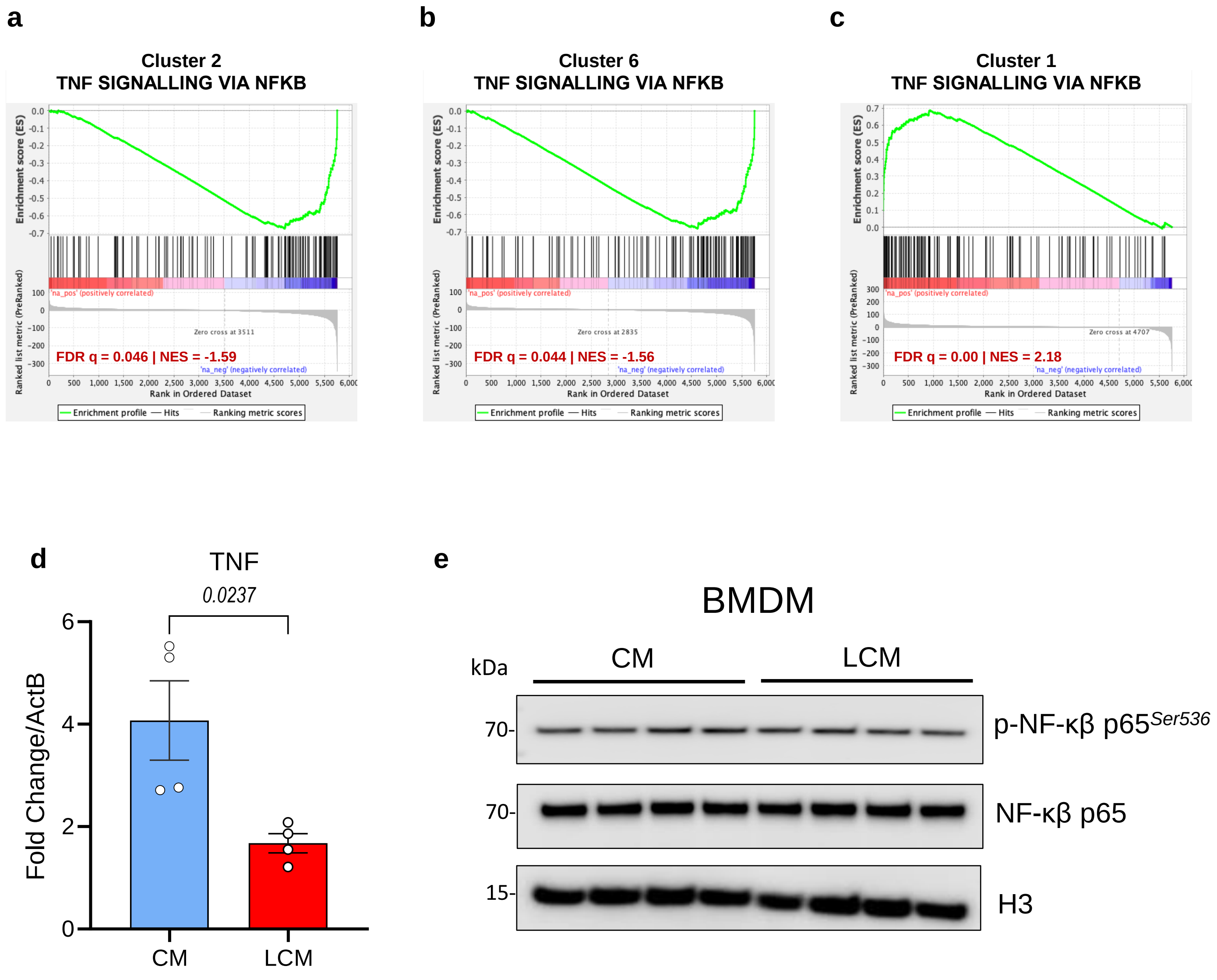

Supplementary Figure 7: CML exposure reduces expression of TNF, but does not alter NF-κβ activity in bone marrow macrophages. **a-c**: Gene set enrichment analysis (GSEA) of cluster 2 (**a**), cluster 6 (**b**) and cluster 1 (**c**). Normalised enrichment score (NES). False discovery rate (FDR). **d**: Relative log fold change in mRNA expression levels in bone marrow derived macrophages (BMDM) conditioned with medium from control (CM) or CML c-Kit+ enriched BM (LCM) for 24hr (n=4 independent experiments). **e**: Immunoblot analysis of NF-κβ phosphorylation in BMDM exposed to CM or LCM for 24hr (n=4 independent cultures). Data are shown as the mean ± s.e.m. P-values were calculated using unpaired two-tailed t-test (**d**).

Supplementary Fig. 8

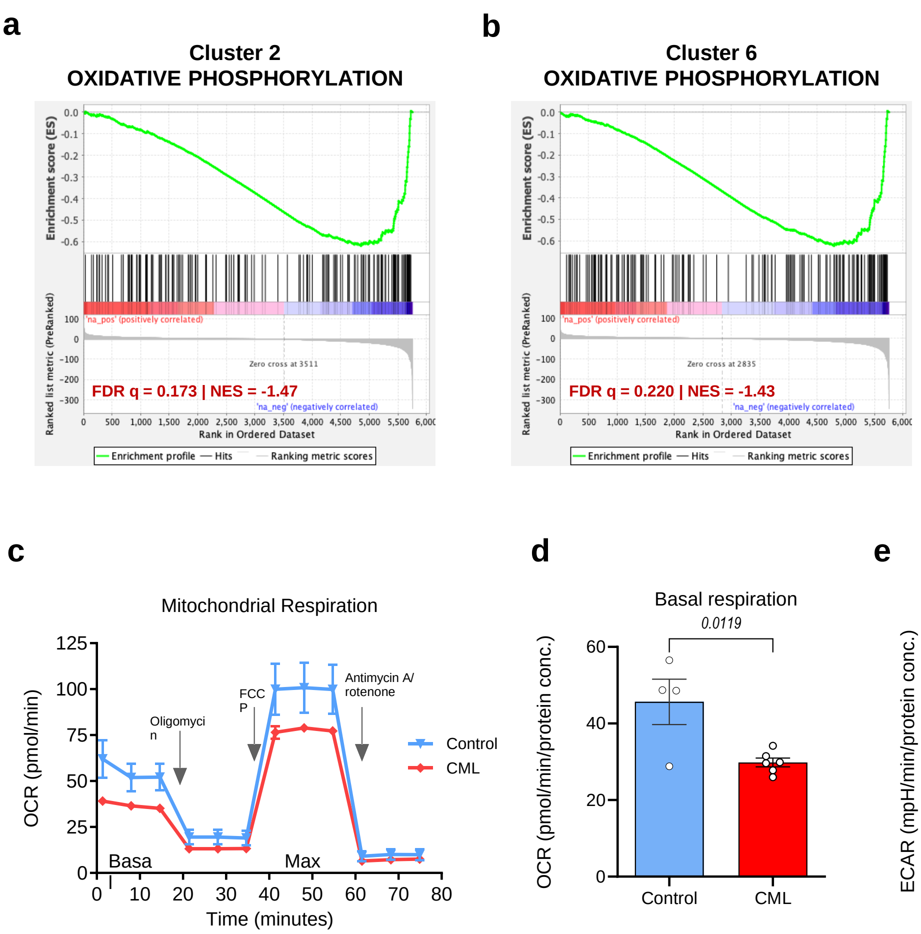

Supplementary Figure 8: CML exposure alters cellular energy in bone marrow macrophages. a-b: GSEA of oxidative phosphorylation in cluster 2 (**a**), cluster 6 (**b**) **d-f**: Representative oxygen consumption rate (OCR) profile (**c**), basal respiration (**d**) and basal glycolysis (**e**) in BMDM following 16hr co-culture with control or CML c-Kit$^+$ cells (n= 4 independent wells for control and n=6 independent wells for CML of one representative experiment, n=2 independent experiments). Data are shown as the mean ± s.e.m. P-values were calculated using unpaired two-tailed t-test (**d, e**).

Supplementary Fig. 9

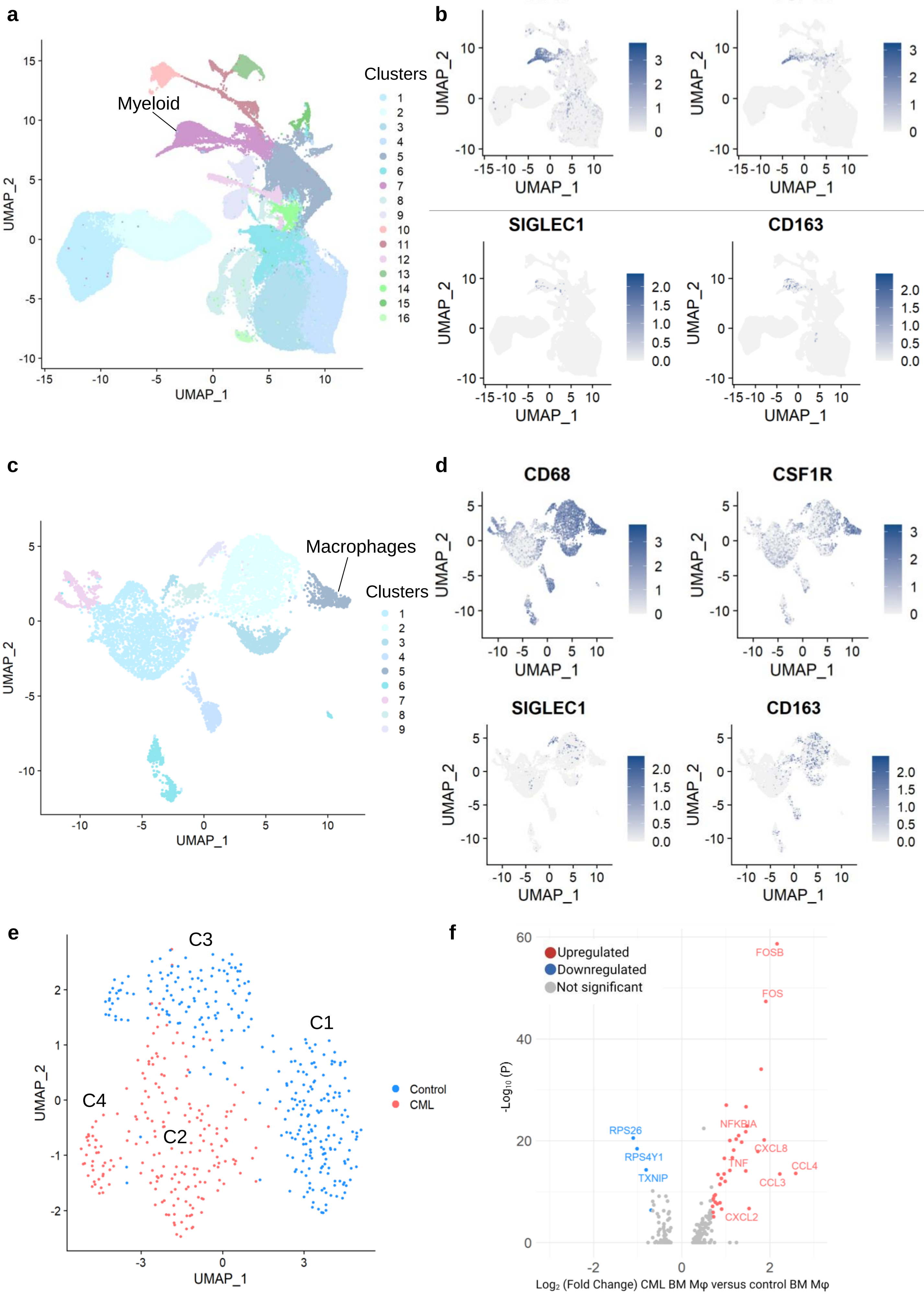

Supplementary Figure 9: Macrophages are transcriptionally altered in BM of CML patients at diagnosis. a: Uniform Manifold Approximation and Projection (UMAP) representation of the pooled RNA profiles of 163,146 single-cells isolated from the BM of CML patients at diagnosis (n=24) or healthy individuals (n=5). **b**: Expression of macrophage markers *CD68*, *CSF1R*, *SIGLEC1* and *CD163* in BM single cells from CML patients at diagnosis or healthy individuals. **c**: UMAP representation of myeloid cluster. **d**: Expression of macrophage markers *CD68*, *CSF1R*, *SIGLEC1* and *CD163* in myeloid cluster. **e**: UMAP representation of macrophage subpopulation according to disease status. **f**: Volcano plot of differentially expressed genes in CML macrophages compared to control macrophages. Upregulated genes with a CML versus control log2 (fold change) of at least 0.7 are shown in red, while downregulated gene with a CML versus control log2 (fold change) of at least -0.7 are shown in blue. P-values were calculated using DESeq2 pairwise two-tailed t-test and the Benjamini-Hochberg method to correct for multiple comparisons.

Supplementary Fig. 10:

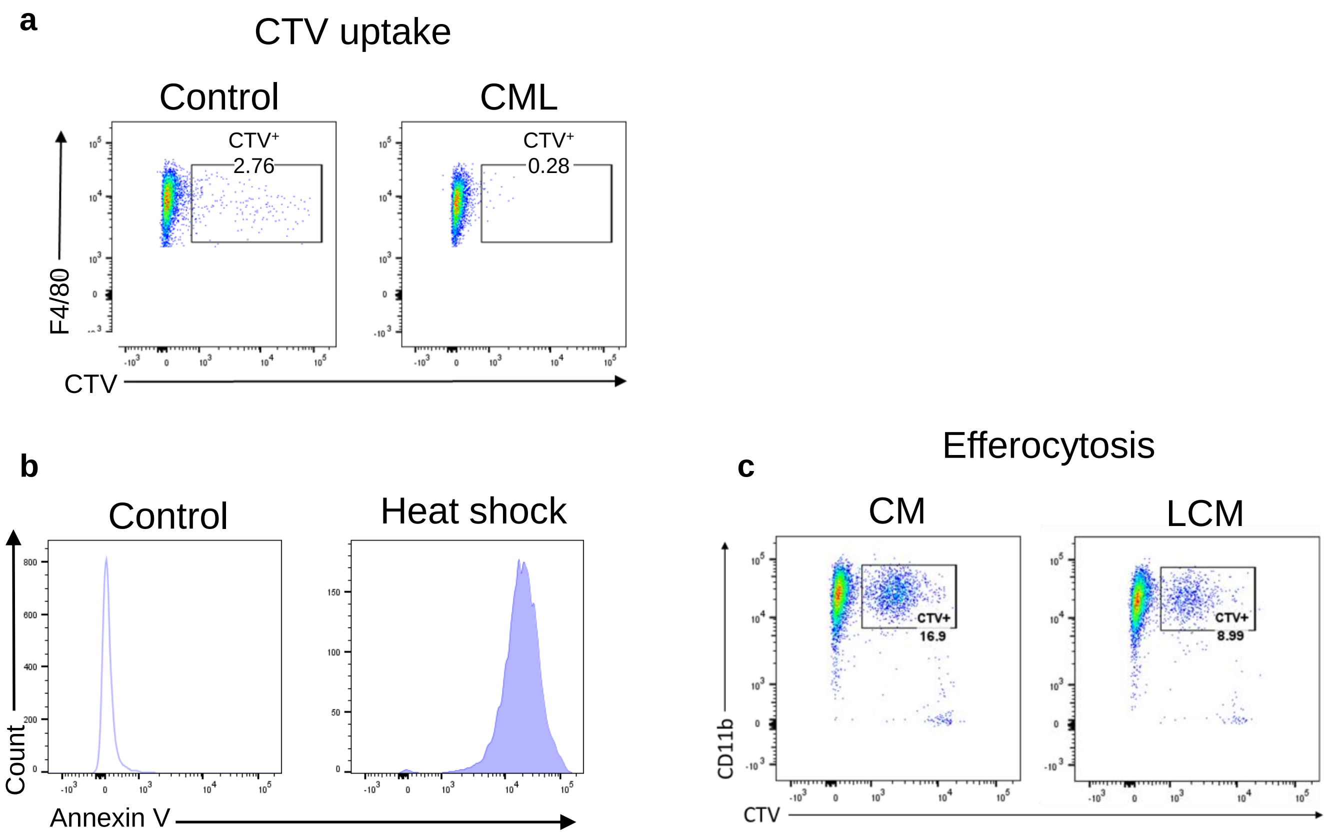

Supplementary Figure 10: CML exposure alters clearance function in mouse macrophages. a: Representative flow cytometry plots of CTV$^+$CD11b$^+$F4/80$^+$ BMDM following Control/CML CTV$^+$ c-Kit$^+$ cells co-culture with BMDM following 48hr culture. b: Representative histogram plots of annexin V staining in K562 cells that were rendered apoptotic by a 45s heat shock at 95ºC. **c**: Flow cytometry plots of CTV/CD11b^{high} BMDM conditioned with CM or LCM for 24hr in the presence of CML apoptotic cells for the final two hours.

Supplementary Fig. 11

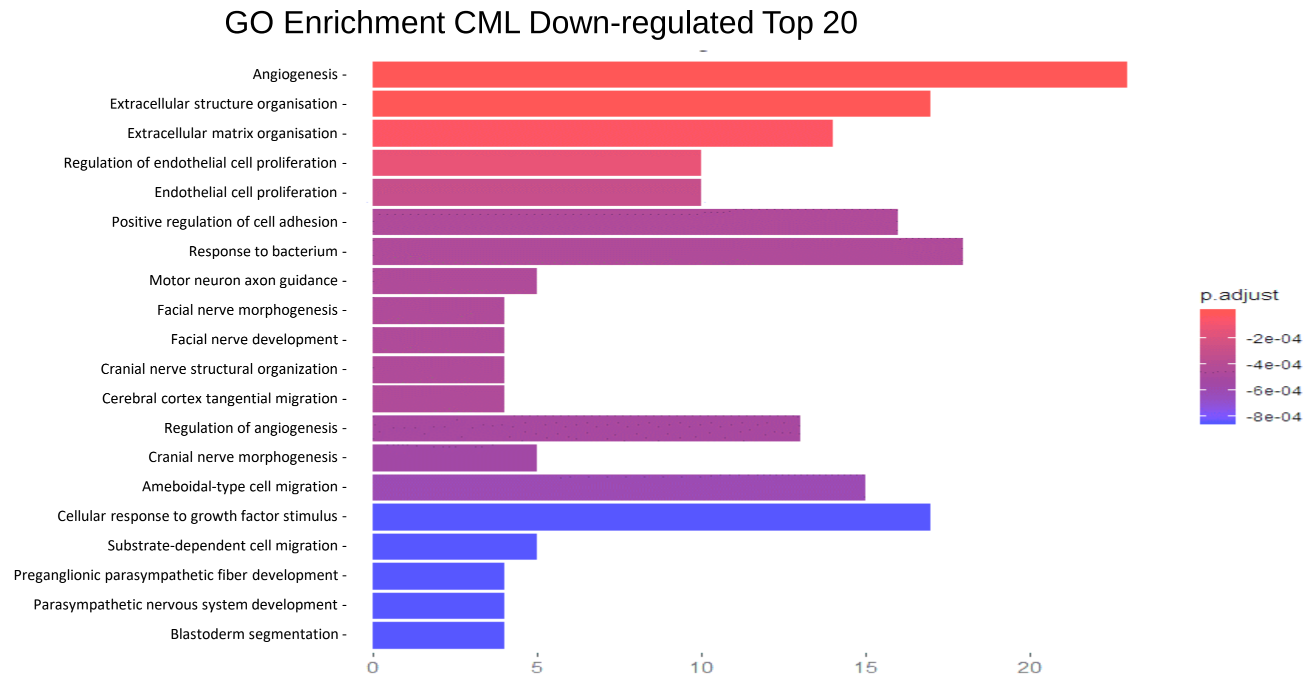

Supplementary Figure 11: Down-regulated pathways in GO enrichment analysis of murine CML LT-HSC RNA Sequencing. GO enrichment analysis of significant differentially expressed genes in CML LT-HSCs. LT-HSC differential expression calculated with DESeq2 pairwise two-tailed t-test and the Benjamini-Hochberg method to correct for multiple comparisons.

Supplementary Fig. 12

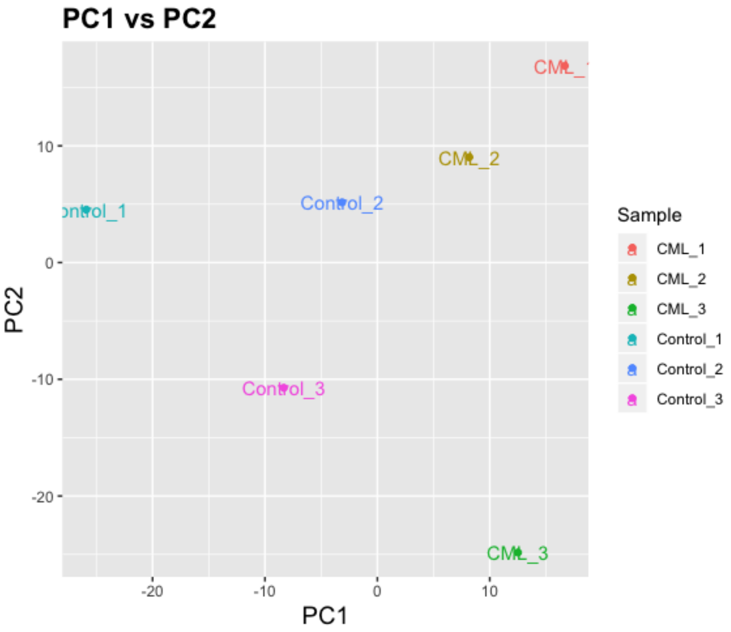

Supplementary Figure 12: Principal component analysis plot of murine c-kit$^+$ cell secretory proteome. Principle component analysis of label free quantification of secreted proteins from murine c-kit$^+$ cells cultured for 24hr. Control mice SCLtTA$^+$/BCR-ABL$^-$ (n=3), CML mice SCLtTA$^+$/BCR-ABL$^+$ (n=3). Data representative of principle component 1 (PC1) vs principal component 2 (PC2).

Supplementary Fig. 13:

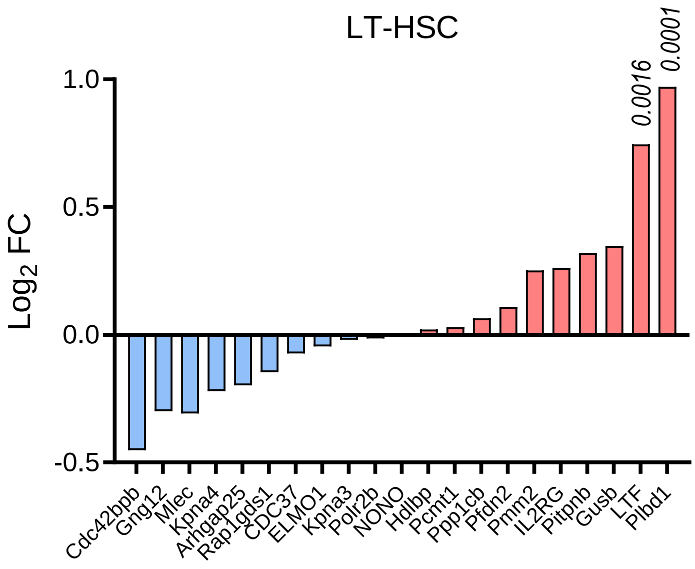

Supplementary Figure 13: Lactotransferrin (LTF) expression is upregulated in CML LT-HSCs. Differential expression of the 20 significantly changed proteins in CML conditioned medium in CML LT-HSC. Differential gene expression analysis was conducted with the DeSeq2 package with the Benjamini-Hochberg method to correct for multiple comparisons.

Supplementary Fig. 14:

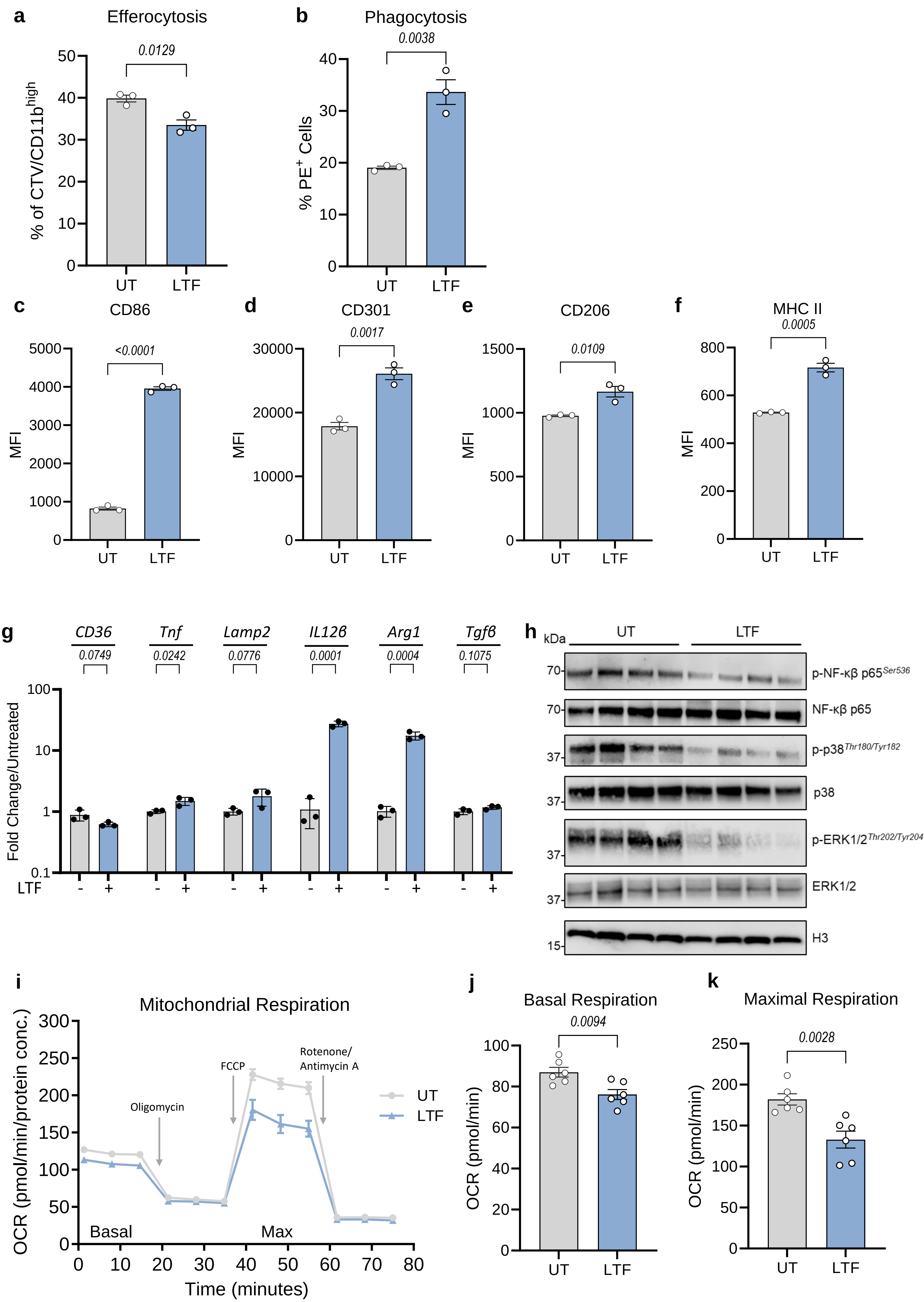

Supplementary Figure 14: LTF exposure alters function and phenotype in murine macrophages. a,b : Quantification of uptake of apoptotic cells (**a**) or latex beads (**b**) in BMDM exposed to 50μg/mL LTF for 24hr (n=3 independent experiments). **c-f**: Surface marker expression of CD86 (**c**), CD301 (**d**), CD206 (**e**) and MHC II (**f**) in BMDM treated with 50μg/mL LTF for 24hr (n=3 independent experiments). **g**: Relative log fold change in mRNA expression levels in BMDM exposed to 50μg/mL LTF for 24hr (n=3 independent experiments). **h**: Western blot analysis of NF-κβ phosphorylation and MAPK signalling in BMDM in the presence or absence of 50μg/mL LTF (n=4 independent cultures). **i-k**: Representative oxygen consumption rate (OCR) profile (**i**) and basal OCR (**j**) and maximum OCR (**k**) in BMDM following 24h LTF treatment (n = 6 independent wells of one representative experiment, n=2 independent experiments). Data are presented as the mean ± s.e.m. P-values were calculated using unpaired two-tailed t-test (**a-j**).

Supplementary Fig. 15:

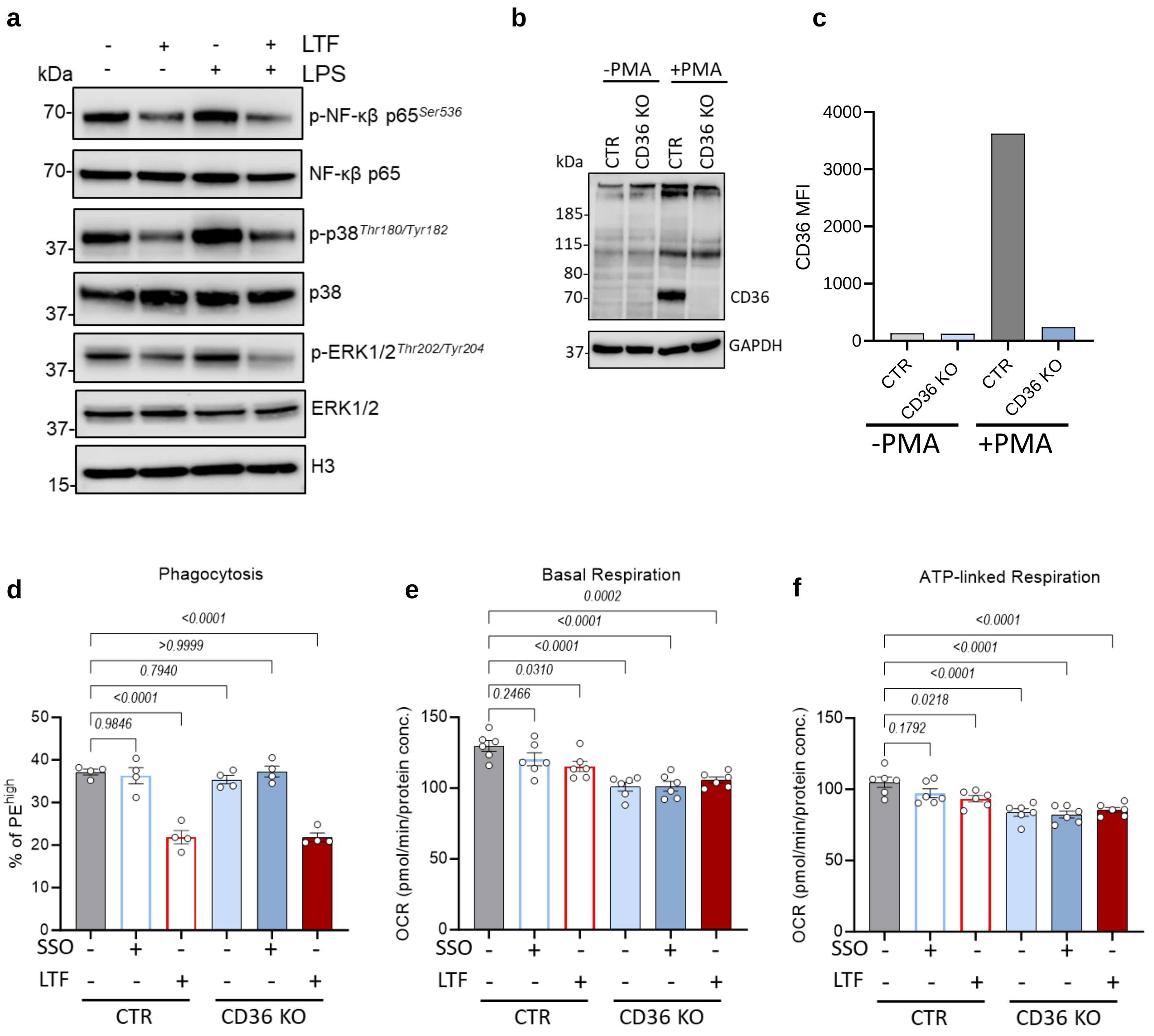

Supplementary Figure 15: Loss of CD36 suppresses mitochondrial respiration in THP-1 derived macrophages. a: Western blot analysis of NF-κB phosphorylation and MAPK signalling in THP1 macrophages treated with 50μg/mL LTF in the presence or absence of 1μg/mL LPS for 24hr (representative of three independent experiments). **b:** Immunoblot analysis of CD36 levels in THP-1 cells expressing an empty vector (CTR) or CD36 knockout (KO) cells in the presence or absence of 20ng/mL phorbol 12-myristate 13-acetate (PMA) for 48h. **c**: Surface expression of CD36 in THP1 CTR or CD36 KO exposed to 20ng/mL PMA for 48hr. **d**: Quantification of phagocytosis of latex beads in THP1 CTR or CD36 KO macrophages treated with 50μM SSO or 50μg/mL LTF for 24hr (n=4 independent experimemts). **e-f**: Representative basal (**e**) and ATP-linked (**f**) respiration in THP1 CTR or CD36 KO macrophages treated with 50μM SSO or 50μg/mL LTF for 24hr (n=6 independent wells of one representative experiment, n=2 independent experiments). Data are shown as the mean ± s.e.m. P-values were calculated using ordinary one-way ANOVA with Dunnet's multiple comparisons test (**d-f**) .

Supplementary Table 1:

Gene	P value	Control (Mean FC)	CML Exposed (Mean FC)
ABCA1	0.33834	229616	24887
CCL2	0.49753	8.616	52.1
CD36	0.00015	1.285	0.006124
CD68	0.71973	5.349	3.91
CD93	0.06266	0.6779	0.001116
CTSB	0.42165	3.458	10.29
CTSD	0.62053	2.283	2.97
CTSS	0.59791	5.298	9.924
EMP1	0.44806	206051	767081
EMR1	0.2775	2.265	9.904
FN1	0.51359	238.3	785.3
GPNMB	0.29182	2607	42.54
HMOX1	0.3945	3.119	7.044
HPRT	0.30436	4.476	42.54
HSP90	0.72897	4.248	5.135
LRP1	0.50362	121560	280467
IL12b	0.30436	4.476	42.54
LAMP	0.03357	0.5743	0.0006272
AXL	0.29272	0.334	0.006376
mEMR1	0.42289	2.639	9.719
MERTK	0.30436	4.476	42.54
MMP12	0.30436	4.476	42.54
Msepp1	0.36008	4.608	31.34
MRC1	0.30436	4.476	42.54
MSR1	0.48989	29106	123358
mTNF	0.98943	1.431	1.426
NOS2	0.30436	4.476	42.54
PDGFC	0.30436	4.476	42.54
PLAU	0.6909	2.832	4.202
PPARG	0.28801	6632	42.54
PSAP	0.6882	7.48	10.95
TGFB1	0.43894	4.804	10.14
TREM2	0.28782	7196	42.54

Supplementary Table 1: Comparative gene expression analysis between WT and CML exposed BM macrophages in vivo. FC, fold change relative to a single WT control. Data representative of mean of n=3 control mice and n=4 CML exposed mice per arm. Statistical analysis: multiple two-tailed T tests with Benjamini-Hochberg method to correct for multiple testing. Significant genes highlighted in red.

Supplementary Table 2:

Gene	P value	CML (Mean FC)	CML Exposed (Mean FC)
ABCA1	0.352042	71022	24887
CCL2	0.35786	2.11	52.1
CD36	0.072511	1.705	0.006124
CD68	0.740626	2.836	3.91
CD93	0.250559	6.737	0.001116
CTSB	0.997443	10.33	10.29
CTSD	0.738914	2.601	2.97
CTSS	0.258017	1.443	9.924
EMP1	0.243748	34116	767081
EMR1	0.277881	3.374	9.904
FN1	0.282367	15.53	785.3
GPNMB	0.09649	7932	42.54
HMOX1	0.189226	1.901	7.044
HPRT	0.24729	36435	42.54
HSP90	0.582174	4.01	5.135
LRP1	0.164446	666.4	280467
IL12b	0.01382	127249	42.54
LAMP	0.076877	0.3272	0.0006272
AXL	0.300221	7.258	0.006376
mEMR1	0.643209	6.188	9.719
MERTK	0.221965	4261	42.54
MMP12	0.212823	2730	42.54
Msepp1	0.224489	1.212	31.34
MRC1	0.167754	30920	42.54
MSR1	0.358741	17139	123358
mTNF	0.663003	1.727	1.426
NOS2	0.068205	2097	42.54
PDGFC	0.3455	9222	42.54
PLAU	0.588174	2.636	4.202
PPARG	0.271994	20955	42.54
PSAP	0.319274	3.796	10.95
TGFB1	0.416602	5.51	10.14
TREM2	0.336011	45491	42.54

Supplementary Table 2: Comparative gene expression analysis between CML and CML exposed BM macrophages in vivo. FC, fold change relative to a single WT control. Data representative of mean of n=4 mice per experimental arm. Statistical analysis: multiple two-tailed T tests with Benjamini-Hochberg method to correct for multiple testing. Significant genes highlighted in red.

Supplementary Table 3:

Supplementary Methods Table 1-Patient information		
ID	Other notes	Figures
CML 1	ELN failure: BCR-ABL 0.11% at 12 months, MMR by 18 months: not resistant	Fig. 7a
CML 2	Failed imatinib -> dasatinib	Fig. 7a
CML 3	ELN warning, responded to imatinib, achieved MMR on dasatinib	Fig. 7a
CML 4	ELN failure: BCR-ABL 1% at 12 months	Fig. 7a

Supplementary Table 3: Patient samples (at diagnosis) used in this study.

-> refers to next treatment

ELN: European LeukaemiaNet (recommendations for the management of CML)

MMR: major molecular response

Supplementary Table 4:

Primers for Fluidigm		
**Gene**		**Primer sequence (5'-3')**
Abca1	Forward	AAAACCGCAGACATCCTTCAG
	Reverse	CATACCGAAACTCGTTCACCC
Anpep	Forward	ATGGAAGGAGGCGTCAAGAAA
	Reverse	CGGATAGGGCTTGGACTCTTT
Arg1	Forward	CTCCAAGCCAAAGTCCTTAGAG
	Reverse	AGGAGCTGTCATTAGGGACATC
Ccl2	Forward	TTAAAAACCTGGATCGGAACCAA
	Reverse	GCATTAGCTTCAGATTTACGGGT
CD36	Forward	AGATGACGTGGCAAAGAACAG
	Reverse	CCTTGGCTAGATAACGAACTCTG
CD68	Forward	CCTCGCCTAGTCCAAGGTC
	Reverse	GGATTCGGATTTGAATTTGGGCT
CD93	Forward	ATCTCAACTGGTTTGTTCCTGC
	Reverse	ACTCTTCACGGTGGCAAGATT
Cts1	Forward	ATCAAACCTTTAGTGCAGAGTGG
	Reverse	CTGTATTCCCCGTTGTGTAGC
Ctsd	Forward	GCTTCCGGTCTTTGACAACCT
	Reverse	CACCAAGCATTAGTTCTCCTCC
Ctss	Forward	CCATTGGGATCTCTGGAAGAAAA
	Reverse	TCATGCCCACTTGGTAGGTAT
Emp1	Forward	TTGGTGCTACTGGCTGGTCT
	Reverse	CATTGCCGTAGGACAGGGAG
Emr1	Forward	TGACTCACCTTGTGGTCCTAA
	Reverse	CTTCCCAGAATCCAGTCTTTCC
Fn1	Forward	TTCAAGTGTGATCCCCATGAAG
	Reverse	CAGGTCTACGGCAGTTGTCA
Gas6	Forward	TGCTGGCTTCCGAGTCTTC
	Reverse	CGGGGTCGTTCTCGAACAC
Gpnmb	Forward	TGCCAAGCGATTTCGTGATGT
	Reverse	GCCACGTAATTGGTTGTGCTC
Hmox1	Forward	AAGCCGAGAATGCTGAGTTCA
	Reverse	GCCGTGTAGATATGGTACAAGGA
Hprt	Forward	TCAGTCAACGGGGACATAAA
	Reverse	GGGGCTGTACTGCTTAACCAG
Hsp90	Forward	GTCCGCCGTGTGTTCATCAT
	Reverse	GCACTTCTTGACGATGTTCTTGC
Igf1	Forward	GCAACACTCATCCACAATGC
	Reverse	AGCTGGACCAGAGACCCTTT
IL12a	Forward	CAATCACGCTACCTCCTCTTTT
	Reverse	CAGCAGTGCAGGAATAATGTTTC
IL12b	Forward	TGGTTTGCCATCGTTTTGCTG
	Reverse	ACAGGTGAGGTTCACTGTTTCT
Lamp2	Forward	TGGCTCAGCTTTCAACATTTCC
	Reverse	TGCCAATTAGGTAAGCAATCACT
Lrp1	Forward	ACTATGGATGCCCCTAAAACTTG
	Reverse	GCAATCTCTTTCACCGTCACA
Axl	Forward	ATGGCCGACATTGCCAGTG
	Reverse	CGGTAGTAATCCCCGTTGTAGA

B2M	Forward	ACCCGCCTCACATTGAAATCC
	Reverse	GGCGTATGTATCAGTCTCAGTG
Ctsb	Forward	TCCTTGATCCTTCTTTCTTGCC
	Reverse	ACAGTGCCACACAGCTTCTTC
Emr1	Forward	TGACTCACCTTGTGGTCCTAA
	Reverse	CTTCCCAGAATCCAGTCTTTCC
Mertk	Forward	CTCCTGAGCCCGTCAATATCT
	Reverse	AGACCAGGTACGGTTAGGACA
lgals3	Forward	GTACAGCTAGCGGAGCGG
	Reverse	CGGATATCCTTGAGGGTTTG
Mmp12	Forward	TTTGGATTATTGGAATGCTGC
	Reverse	ATGAGGCAGAAACGTGGACT
Mmp2	Forward	CAAGTTCCCCGGCGATGTC
	Reverse	TTCTGGTCAAGGTCACCTGTC
Mmp3	Forward	TCTGGGCTATACGAGGGCAC
	Reverse	ACCCTTGAGTCAACACCTGGA
Mrc1	Forward	CTCTGTTCAGCTATTGGACGC
	Reverse	CGGAATTTCTGGGATTCAGCTTC
Sepp1	Forward	GGGGCTTTGTAACAAGCAGA
	Reverse	GCAAAGAGACAGGACGAAGC
Msr1	Forward	TTCAAACTCAAAAGCCGACCT
	Reverse	GTTGCCATGCTGAAATTCTGG
Tnf	Forward	CAGGCGGTGCCTATGTCTC
	Reverse	CGATCACCCCGAAGTTCAGTAG
Nos2	Forward	ACATCGACCCGTCCACAGTAT
	Reverse	CAGAGGGGTAGGCTTGTCTC
PDGFC	Forward	ACATTTGATGAGAGATTTGGGCT
	Reverse	CAGCGTCCTAAAACACTTCCAT
Plau	Forward	GCGCCTTGGTGGTGAAAAAC
	Reverse	TTGTAGGACACGCATACACCT
Pparg	Forward	GGAAGACCACTCGCATTCCTT
	Reverse	GTAATCAGCAACCATTGGGTCA
Psap	Forward	CCTGTCCAAGACCCGAAGAC
	Reverse	CAAGGAAGGGATTTCGCTGTG
Tgfb1	Forward	CTTCAATACGTCAGACATTCGGG
	Reverse	GTAACGCCAGGAATTGTTGCTA
Timp2	Forward	TCAGAGCCAAAGCAGTGAGC
	Reverse	GCCGTGTAGATAAACTCGATGTC
Trem2	Forward	CTGGAACCGTCACCATCACTC
	Reverse	CGAAACTCGATGACTCCTCGG
18s	Forward	GTA ACC CGT TGA ACC CCA TT
	Reverse	CCA TCC AAT CGG TAG TAG CG

Supplementary Table 4: Primers for Fluidigm multiplex RT-qPCR

Full western blot membranes

Supplementary Figure 7e:

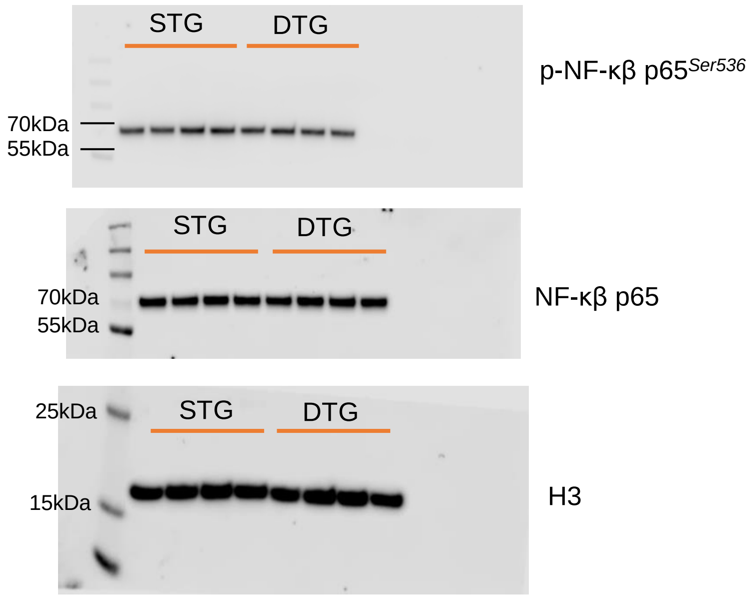

Supplementary Figure 14h:

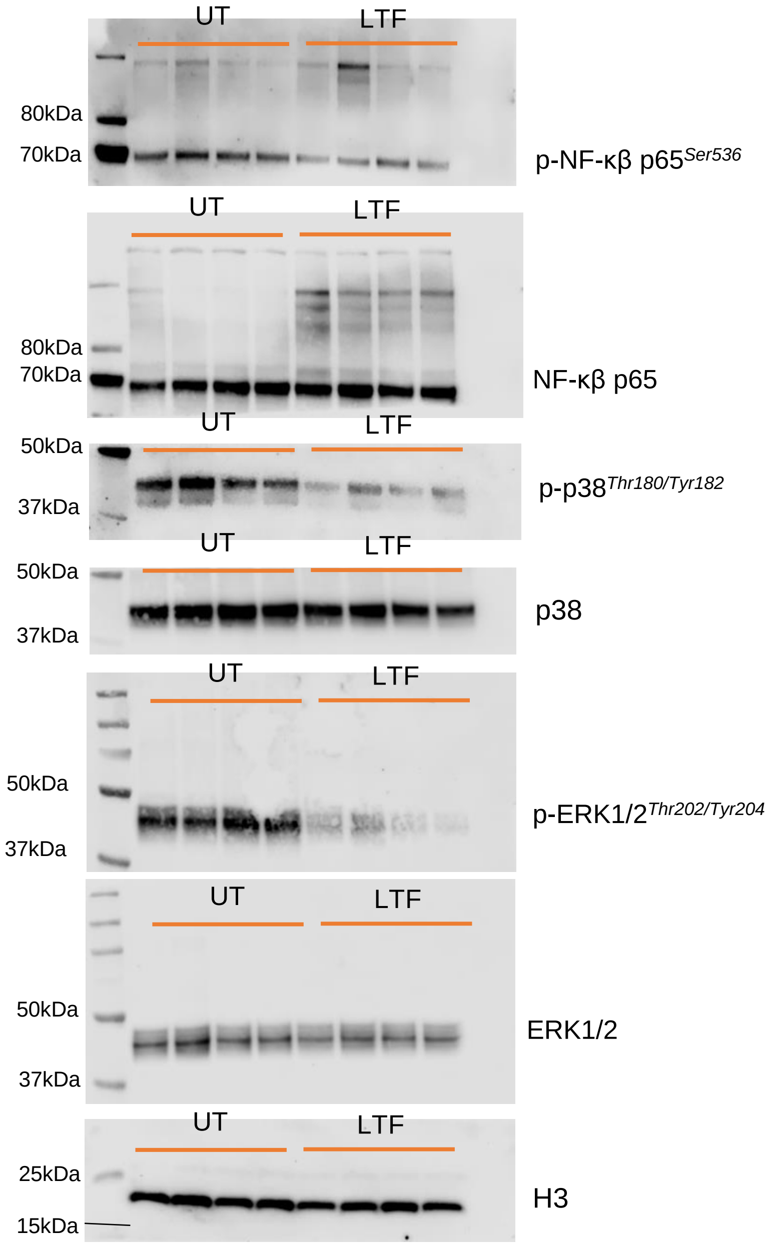

Supplementary Figure 15a:

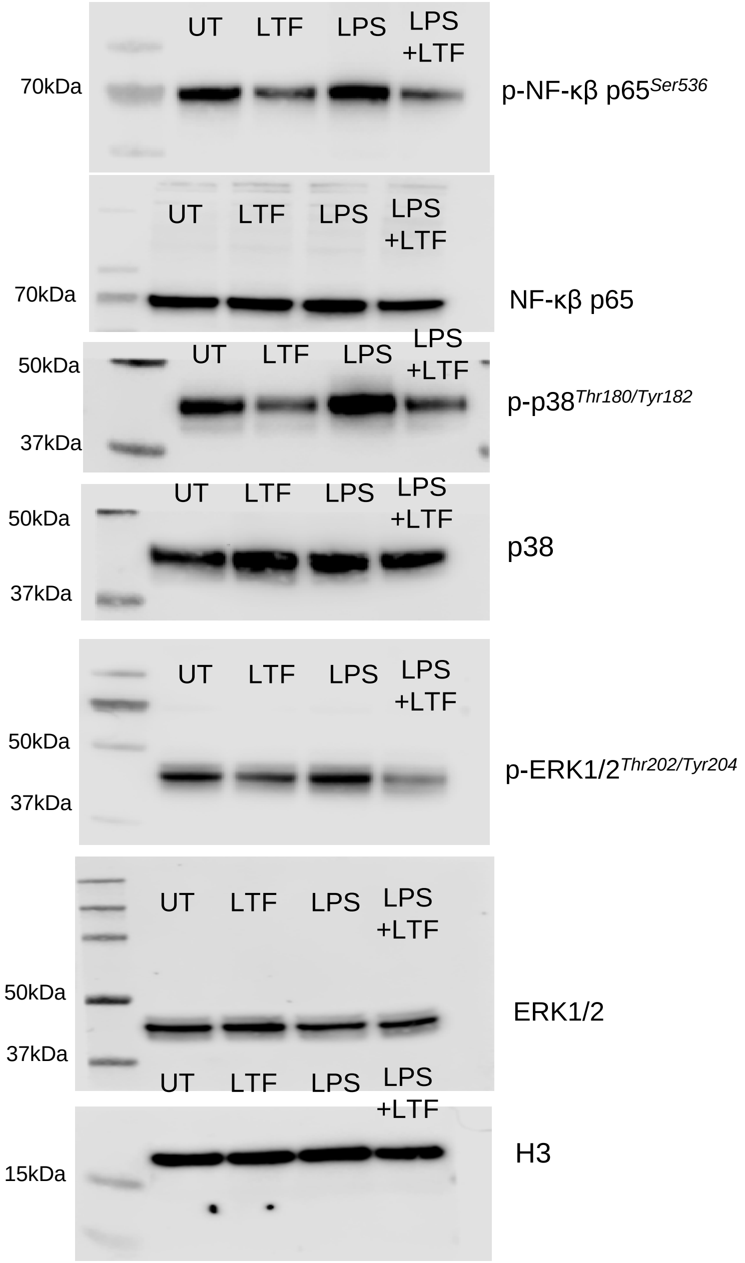

Supplementary Figure 15b:

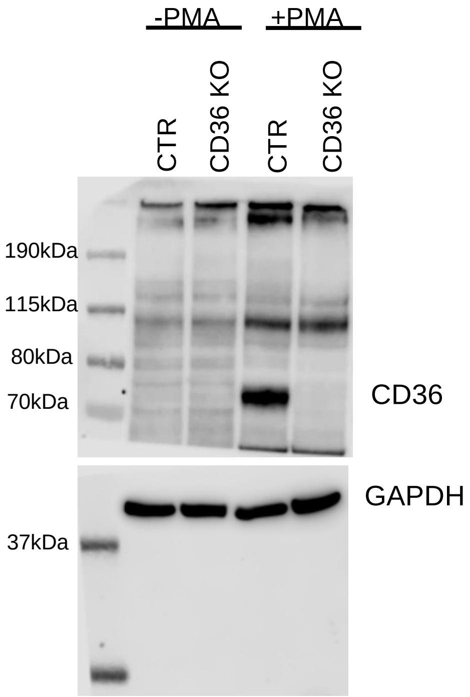

Supplement: Supplementary file 1 — Supplementary Information [file 41467_2024_45471_MOESM1_ESM.pdf]
