# Supplementary material for: Leukaemia exposure alters the transcriptional profile and function of BCR::ABL1 negative macrophages in the bone marrow niche
Source: Nat Commun. 2024 Feb 5;15:1090. doi: 10.1038/s41467-024-45471-0 (PMC10844594; doi:10.1038/s41467-024-45471-0)
Supplement: Supplementary file 3 — Reporting Summary [file 41467_2024_45471_MOESM3_ESM.pdf]

nature portfolio

Corresponding author(s): Kristina Kirschner, G. Vignir Helgason,

Last updated by author(s): Jan 2, 2024

Reporting Summary

Nature Portfolio wishes to improve the reproducibility of the work that we publish. This form provides structure for consistency and transparency in reporting. For further information on Nature Portfolio policies, see our Editorial Policies and the Editorial Policy Checklist.

Statistics

For all statistical analyses, confirm that the following items are present in the figure legend, table legend, main text, or Methods section.

n/a	Confirmed	
☐	☒	The exact sample size (*n*) for each experimental group/condition, given as a discrete number and unit of measurement
☐	☒	A statement on whether measurements were taken from distinct samples or whether the same sample was measured repeatedly
☐	☒	The statistical test(s) used AND whether they are one- or two-sided<br>*Only common tests should be described solely by name; describe more complex techniques in the Methods section.*
☐	☒	A description of all covariates tested
☐	☒	A description of any assumptions or corrections, such as tests of normality and adjustment for multiple comparisons
☐	☒	A full description of the statistical parameters including central tendency (e.g. means) or other basic estimates (e.g. regression coefficient) AND variation (e.g. standard deviation) or associated estimates of uncertainty (e.g. confidence intervals)
☐	☒	For null hypothesis testing, the test statistic (e.g. *F*, *t*, *r*) with confidence intervals, effect sizes, degrees of freedom and *P* value noted<br>*Give P values as exact values whenever suitable.*
☒	☐	For Bayesian analysis, information on the choice of priors and Markov chain Monte Carlo settings
☐	☒	For hierarchical and complex designs, identification of the appropriate level for tests and full reporting of outcomes
☒	☐	Estimates of effect sizes (e.g. Cohen's *d*, Pearson's *r*), indicating how they were calculated

Our web collection on statistics for biologists contains articles on many of the points above.

Software and code

Policy information about availability of computer code

Data collection	For flow cytometry, stained cells were collected by FACSVerseTM Flow Cytometer (BD Biosciences) or BD FACS ARIA Z6001 (BD Biosciences). For mass spectrometry, spectra were acquired in the Orbitrap analyser. Sequencing was performed using the NextSeq500 platform with the HiSeq SBS Kit (v.4) (Illumina). For PCR analysis data were acquired with the C1000 Touch Thermal Cycler (BioRad) and CFX Maestro™ Software (v.1.1) (BioRad). For western blots, images were acquired with the LI-COR Odyssey FC imaging (LI-COR Biosciences) system with Image Studio software (v.5.2). For metabolic analysis of macrophages, data were acquired using the XF96 flux analyzer (Seahorse Bioscience).
Data analysis	Flow cytometry data was analyzed using FlowJo (v.10.7). For mass spectrometry analysis, tryptic peptides were analysed on Q-Exactive HF (Thermo Scientific) coupled online to an EASY-nLC II (Thermo Scientific). The mass spectrometry files were analysed using MaxQuant software (v.1.5.51) and searched against the mouse UniProt database for protein identification. For single cell bioinformatic data analysis, sequences were checked for quality using FastQC v0.11.8 and trimmed with Trim_galore v0.5.0. Sequences were aligned to the reference genome (GRCm38, obtained via Ensembl on 10/12/2017 with ERCC sequence - Thermo-Fischer, #4456739 - manually added) using Hisat2 v2.1.0. A count matrix was produced with FeatureCounts v1.6.2. Further data analysis was performed using Scater and Scran packages in R software. T-SNE plots were produced using Scater. Markers for clusters were obtained using the findMarkers function in Scran and heatmaps were plotted using Scater. Violin plots were generated using ggplot2. Pathway analysis using Kegg, Reactome and Wikipathways was performed using http://www.webgestalt.org. For RNA sequencing/gene ontogeny (GO) analysis the clusterProfiler package with the enrichGO function was used to determine significantly enriched GO terms. Statistical analysis was performed using R Studio (v.1.1.4). For the human scRNA-seq dataset, data analysis was performed using Seurat66 package in R (v.4.2.2). Statistical analysis was performed using GraphPad Prism 9 Software (v.9.3.1). For immunoblotting, image analysis was conducted using mage Studio software (v.5.2).

For manuscripts utilizing custom algorithms or software that are central to the research but not yet described in published literature, software must be made available to editors and reviewers. We strongly encourage code deposition in a community repository (e.g. GitHub). See the Nature Portfolio guidelines for submitting code & software for further information.

Data

Policy information about availability of data

All manuscripts must include a data availability statement. This statement should provide the following information, where applicable:

- Accession codes, unique identifiers, or web links for publicly available datasets
- A description of any restrictions on data availability
- For clinical datasets or third party data, please ensure that the statement adheres to our policy

The expression profiling scRNA-seq data generated in this study have been deposited in public Gene Expression Omnibus (GEO) database under accession code GSE204978. Data request can also be made by contacting the corresponding authors (vignir.helgason@glasgow.ac.uk or kristina.kirschner@glasgow.ac.uk). Raw data used in Figure 5a-b and Supplementary Figure 11 has been uploaded to GEO under accession code GSE12654771 and is available to the scientific community/ general public. Data request, including relevant codes, can also be made by contacting co-author Ravi Bhatia (rbhatia@uabmc.edu). Raw data used in figure 5d-e has been deposited to the ProteomeXchange Consortium via the PRIDE partner repository72. Dataset identifier is PXD022366. Raw data used in Supplementary Figure 9 are available online at the European Genome-Phenome Archive (EGA) under accession ID EGAS00001005509 upon written request to the data access committee. Using a R shiny APP, the scRNA-seq data can be interactively viewed at: http://scdbm.ddnetbio.com/. Data request, including relevant code can also be made by contacting co-author Ong Sing Tiong (sintiong.ong@duke-nus.edu.sg). The remaining data are available within the Article, Supplementary Information or Source Data file.

Research involving human participants, their data, or biological material

Policy information about studies with human participants or human data. See also policy information about sex, gender (identity/presentation), and sexual orientation and race, ethnicity and racism.

Reporting on sex and gender	In this study, equal number of male (2) and female (2) patient samples was used. The median age at diagnosis of patients was 58.5 years old, with ages ranging from 27-63 years old
Reporting on race, ethnicity, or other socially relevant groupings	This data was not collected.
Population characteristics	Limited data on follow-up/current status was obtained with the exception being treatment failure. Data are available on Supplementary mehods table 1.
Recruitment	Authors had no role in the original collection of samples.
Ethics oversight	All patient samples were kindly donated with ethical approval and informed consent in agreement with the Declaration of Helsinki and with the approval of the National Health Service (NHS) Greater Glasgow and Clyde Institutional Review Board. Ethical approval was granted to the research tissue bank (REC 15/WS/0077) and for using surplus human tissue in research (REC 10/S0704/60).

Note that full information on the approval of the study protocol must also be provided in the manuscript.

Field-specific reporting

Please select the one below that is the best fit for your research. If you are not sure, read the appropriate sections before making your selection.

☒ Life sciences ☐ Behavioural & social sciences ☐ Ecological, evolutionary & environmental sciences

For a reference copy of the document with all sections, see nature.com/documents/nr-reporting-summary-flat.pdf

Life sciences study design

All studies must disclose on these points even when the disclosure is negative.

Sample size	No statistical methods were used to predetermine sample size for in vitro experiments. Sample sizes were estimated according to common practice for each experimental design and preliminary and previous experiments to estimate variability. In vitro experiments were repeated at least on three separate occasions, except where noted. For experiments with primary samples, a minimum of four samples was used to give adequate power. For in vivo experiments, while no statistical methods were used to calculate the exact cohort sample size, the number of animals used per arm in each experiment was estimated based on variability of pilot and previous experiments. he number of biological replicates and applied statistical analysis are indicated in the figure legends. No relevant data was excluded in the analysis.
Data exclusions	No relevant data was excluded in the analysis.
Replication	Experiments were repeated as detailed in the figure legends. This was 3-4 independent replicates with exact numbers provided in figure legeneds.
Randomization	For all in vitro experiments, unless stated otherwise, cells were randomly plated/treated/analysed during each experiment. For in vivo experiments, animals were assigned to different groups in a manner ensuring a consistent average starting engraftment across the groups.
Blinding	Investigators were not blinded during data processing and analysis of in vitro data. For collection of in vivo data, investigators were not

blinded to group allocation as the experimental design made this not feasible. For data analysis, investigators were blinded to the experimental conditions when assessing the outcomes, if feasible.

Reporting for specific materials, systems and methods

We require information from authors about some types of materials, experimental systems and methods used in many studies. Here, indicate whether each material, system or method listed is relevant to your study. If you are not sure if a list item applies to your research, read the appropriate section before selecting a response.

Materials & experimental systems

n/a	Involved in the study
☐	☒ Antibodies
☐	☒ Eukaryotic cell lines
☒	☐ Palaeontology and archaeology
☐	☒ Animals and other organisms
☐	☒ Clinical data
☒	☐ Dual use research of concern
☒	☐ Plants

Methods

n/a	Involved in the study
☒	☐ ChIP-seq
☐	☒ Flow cytometry
☒	☐ MRI-based neuroimaging

Antibodies

Antibodies used

For flow cytometry, Cell suspensions were prepared from the BM isolated from the hind limbs of chimeric mice in 2% FBS/PBS solution. Cells were stained (100µL/test) with monoclonal antibodies to mouse CD45.1-FITC (clone A20, Cat# 110705, RRID: AB_313495, Biolegend, 0.5 µL), CD45.1-PE (clone A20, Cat# 110707, RRID: AB_313496, Biolegend, 0.5 µL) CD45.2-PB (clone 104, Cat# 109820, RRID: AB_492873, Biolegend, 0.5 µL), CD45.2-PerCp/Cy5.5 (clone 104, Cat# 109827, RRID: AB_89335, Biolegend, 0.5 µL), CD11b-PE (clone M1/70, Cat# 101208, RRID: B_312791, Biolegend, 0.5 µL) , Biotin-CD11b (clone M1/70, Cat# 553309, RRID: AB_394773, BD Biosciences, 0.5µL), F4/80-APC/Cy7 (clone BM8, Cat# 123117, RRID: AB_893489, Biolegend, 0.5µL), Ly6G-APC (clone 1A8, Cat# 127613, RRID: AB_1877163, Biolegend, 0.5µL), Ly6G-PB (clone 1A8, Cat# 127612, RRID: AB_2251161, Biolegend, 0.5µL), CD3-APC (clone 17A2, Cat# 100235, RRID: AB_2561456, Biolegend, 0.5µL), CD4-PE (clone GK1.5, Cat# 100408, RRID: AB_312693, 0.5µL), CD8a-PE (Clone 53-6.7, Cat# 100707, RRID: AB_312746, 0.5µL), CD19-APC/Cy7 (clone 6D5, Cat# 115530, RRID: AB_830707, Biolegend, 0.5µL), Lineage cocktail-PB (clone 17A2; RB6-8C5; RA3-6B2; Ter-119; M1/70, Cat# 133310, RRID: AB_11150779, Biolegend, 10µL), SCA-1-Pe/Cy7 (clone D7, Cat# 108114, RRID: AB_493596, 1µL) CD117 (c-Kit)-APC/Cy7 (clone 2B8, Cat# 105812, RRID: AB_313221, Biolegend, 1µL), CD48-PE (clone HM48-1, Cat# 103406, RRID: AB_313021, Biolegend, 1µL), CD150-APC (clone TC15-12F12.2, Cat# 115910, RRID: AB_493460, Biolegend, 1µL) and CD11c-PE/Cy7 (clone N418, Cat# 117318, RRID: AB_493568, Biolegend, 1µL).

Following exposure to indicated treatments, mouse BMDM were lifted and stained (100µL/test) with monoclonal antibodies to mouse CD11b-PE (clone M1/70, Cat# 101208, RRID: B_312791, Biolegend, 0.5µL), CD36-PE/Cy7 (clone HM36, Cat# 102616, RRID: AB_2566122, Biolegend, 0.5µL), CD86-APC (clone GL-1, Cat# 105011, RRID: AB_493342, Biolegend, 0.5µL), CD206-AlexaFluor488 (clone C068C2, Cat# 141710, RRID: AB_10900445, Biolegend, 0.5µL), CD301-PE/Cy7 (clone LOM-14, Cat# 145705, RRID: AB_2562940, Biolegend, 0.5µL) and MHC II-PerCP/Cy5.5 (clone AF6-120.1, Cat# 116415, RRID: AB_1953308, Biolegend, 0.5µL) .

Similarly, human BMDM or THP-1 macrophages were stained with human CD11b-PE (clone M1/70, Cat# 101208, RRID: B_312791, Biolegend, 0.5µL) and/or CD36-PE/Cy7 (clone 5-271, Cat# 336222, RRID: AB_2716142, Biolegend, 0.5µL).

For western blot, the following primary antibodies were used: Phospho-NF-κB p65 (Ser536) (Clone 93H1, Cat# 3033, Cell Signalling Technology, 1:1000), NF-κB p65 (Clone D14E12, Cat# 8242, Cell Signalling Technology, 1:1000), phospho-p38 MAPK (Thr180/Tyr182) (Clone D3F9, Cat# 4511, Cell Signalling Technology, 1:2000), p38 MAPK (Clone D13E1, Cat# 8690, Cell Signalling Technology, 1:2000), phospho-p44/42 MAPK (Erk1/2) (Thr202/Tyr204) (Clone D13.14.4E, Cat# 4370, Cell Signalling Technology, 1:2000), p44/42 MAPK (Erk1/2) (Clone 137F5, Cat# 4695, Cell Signalling Technology, 1:2000), CD36 (Clone EPR6573, Cat# ab133625, Abcam, 1:1000), H3 (Clone D1H2, Cat# 4499, Cell Signalling Technology, 1:2000) and GAPDH (Clone D16H11, Cat# 5174, Cell Signalling Technology, 1:2000). The following day, membranes were incubated with rabbit HRP-linked secondary antibody (Cat# 7074, Cell Signalling Technology, 1:3000)

Validation

CD45.1-FITC: validated by manufacturer on mouse splenocytes
CD45.1-PE: validated by manufacturer on mouse splenocytes
CD45.2-PB: validated by manufacturer on mouse splenocytes
CD45.2-PerCp/Cy5.5: validated by manufacturer on mouse splenocytes
CD11b-PE: validated by manufacturer on mouse bone marrow cells alongside isotope control
Biotin-CD11b: validated by manufacturer on mouse bone marrow myeloid cells alongside isotope control
F480-APC/Cy7: validated by manufacturer on mouse peritoneal macrophages alongside isotope control
Ly6G-APC: validated by manufacturer on mouse bone marrow cells alongside isotope control
Ly6G-PB: validated by manufacturer on mouse bone marrow cells
CD3-APC: validated by manufacturer on mouse splenocytes
CD4-PE: validated by manufacturer on mouse splenocytes alongside isotope control
CD8a-PE: validated by manufacturer on mouse splenocytes alongside isotope control
CD19-APC/Cy7: validated by manufacturer on mouse splenocytes alongside isotope control

Lineage cocktail-PB: validated by manufacturer on mouse bone marrow cells alongside isotope control
SCA-1-PE/Cy7: validated by manufacturer on mouse splenocytes
CD117-APC/Cy7: validated by manufacturer on mouse splenocytes
CD48-PE: validated by manufacturer on mouse splenocytes
CD150-APC: validated by manufacturer on mouse splenocytes alongside isotope control
CD11c-PE/Cy7: validated by manufacturer on mouse splenocytes alongside isotope control
CD36-PE/Cy7: validated by manufacturer on mouse peritoneal macrophages alongside isotope control
CD86-APC: validated by manufacturer on mouse splenocytes
CD206-AlexaFluor488: validated by manufacturer on mouse peritoneal macrophages alongside isotope control
CD301-PE/Cy7: validated by manufacturer on bone marrow dendritic cells alongside isotope control
MHC II-PerCP/Cy5.5: validated by manufacturer on mouse splenocytes alongside isotope control
human CD36-PE/Cy7: validated by manufacturer on human peripheral blood platelets alongside isotope control
Phospho-NF-κB p65 (Ser536): validated by manufacturer on extracts from HeLa and NIH/3T3 cells, untreated or TNF-α treated
NF-κB p65: validated by manufacturer on various cell lines
phospho-p38 MAPK (Thr180/Tyr182): validated by manufacturer on extracts from COS and 293 cells, untreated or UV-treated
p38 MAPK: validated by manufacturer in various cell lines
phospho-p44/42 MAPK (Erk1/2) (Thr202/Tyr204) : validated by manufacturer on extracts from COS cells, untreated or treated with either U0126 (10 µM for 1h) or TPA (200 nM for 10 m)
p44/42 MAPK (Erk1/2): validated by manufacturer in various cell lines
CD36: validated by manufacturer in THP-1 PMA treated or untreated cells and validated here in THP1 CD36 KO cells
H3: validated by manufacturer in various cell lines and used routinely in host lab
GAPDH: validated in various cell lines by the manufacturer

Eukaryotic cell lines

Policy information about cell lines and Sex and Gender in Research

Cell line source(s)	THP-1, K562, HEK 293FT cell lines were originally purchased from American Type Culture Collection and cultured following the manufacturer's instructions.
Authentication	THP-1, K562, HEK 293FT cell lines were authenticated using short-tandem repeat (STR) profiling
Mycoplasma contamination	THP-1, K562, HEK 293FT cell line were tested negative in-house for mycoplasma contamination
Commonly misidentified lines (See ICLAC register)	none

Animals and other research organisms

Policy information about studies involving animals; ARRIVE guidelines recommended for reporting animal research, and Sex and Gender in Research

Laboratory animals	Previously generated (Koschmieder, S. et al, Blood, 2005) CML mouse model (BCR-ABL/SCL-tTA) was used for in vivo work. Bone marrow was isolated from the hips and hind limbs of wild type (WT) C57/Bl6 mice (Charles River laboratories) through manual crushing. To generate a chimeric model, a 50:50 ratio of CD45.1 WT C57/Bl6 bone marrow was combined with CD45.2 control bone marrow and transplanted in WT C57/Bl6 mice, 8-12 weeks old. Animals of both sexes were housed in a pathogen-free facility at the Cancer Research UK Scotland Institute and kept in day/night cycles (12 hours each), with temperature of 20–24°C and humidity 45–65%. Mice were fed with ad libitum with food and water.
Wild animals	No wild animals were used in this study
Reporting on sex	Sex of mice was not considered. Equal number of male and female mice was used when feasible. Sex of mice was not considered during analysis, and this data have not been collected or included in the study.
Field-collected samples	No field collected samples were used in this study.
Ethics oversight	All mouse experiments were performed in accordance with Home Office regulations and under approved project licences PPL PP2518370, PD6C67A47 and personal licences PIL IC9AB0748, I1F599357.

Note that full information on the approval of the study protocol must also be provided in the manuscript.

Clinical data

Policy information about clinical studies
All manuscripts should comply with the ICMJEguidelines for publication of clinical research and a completedCONSORT checklist must be included with all submissions.

Clinical trial registration	N/A
Study protocol	N/A

Data collection	N/A
Outcomes	N/A

Plants

Seed stocks	*Report on the source of all seed stocks or other plant material used. If applicable, state the seed stock centre and catalogue number. If plant specimens were collected from the field, describe the collection location, date and sampling procedures.*
Novel plant genotypes	*Describe the methods by which all novel plant genotypes were produced. This includes those generated by transgenic approaches, gene editing, chemical/radiation-based mutagenesis and hybridization. For transgenic lines, describe the transformation method, the number of independent lines analyzed and the generation upon which experiments were performed. For gene-edited lines, describe the editor used, the endogenous sequence targeted for editing, the targeting guide RNA sequence (if applicable) and how the editor was applied.*
Authentication	*Describe any authentication procedures for each seed stock used or novel genotype generated. Describe any experiments used to assess the effect of a mutation and, where applicable, how potential secondary effects (e.g. second site T-DNA insertions, mosiacism, off-target gene editing) were examined.*

Flow Cytometry

Plots

Confirm that:

- [x] The axis labels state the marker and fluorochrome used (e.g. CD4-FITC).
- [x] The axis scales are clearly visible. Include numbers along axes only for bottom left plot of group (a 'group' is an analysis of identical markers).
- [x] All plots are contour plots with outliers or pseudocolor plots.
- [x] A numerical value for number of cells or percentage (with statistics) is provided.

Methodology

Sample preparation	Cell suspensions were prepared from the bone marrow isolated from the hind limbs of chimeric mice in 2% FBS/PBS solution. Cells were stained with monoclonal antibodies to mouse CD45.1, CD45.2, CD11b, F4/80, Ly6G, CD3, CD4, CD8a, CD19, Lineage cocktail, SCA-1, c-Kit, CD48, CD150 (Biolegend/BD Biosciences. Following exposure to indicated treatments, mouse BMDM were lifted and stained with monoclonal antibodies to mouse CD11b, CD36, CD86, CD206 and CD301. Similarly, human BMDM or THP-1 macrophages were stained with human CD11b and/or CD36. Stained cells were analysed on FACSVerseTM Flow Cytometer (BD Biosciences). Data represented as percentage of whole bone marrow and absolute cell number calculated from whole bone marrow counts.
Instrument	FACSVerseTM Flow Cytometer (BD Biosciences).
Software	FACSVerseTM Flow Cytometer (BD Biosciences). Flow cytometry data was analyzed using FlowJo (v.10.9)
Cell population abundance	N/A
Gating strategy	For all flow cytometry assays, cells were firstly gated by FSC/SSC to exclude debris. Gating strategy is shown in supplementary data file.

- [x] Tick this box to confirm that a figure exemplifying the gating strategy is provided in the Supplementary Information.
